# Electrochemical oxidizing digestion using PbO_2_ electrode for total phosphorus determination in a water sample

**DOI:** 10.1039/c8ra00220g

**Published:** 2018-02-07

**Authors:** Tong Qi, Ziqi Su, Yan Jin, Yuqing Ge, Hui Guo, Hui Zhao, Jiaqiang Xu, Qinghui Jin, Jianlong Zhao

**Affiliations:** NEST Lab, Department of Chemistry, College of Sciences, Shanghai University Shanghai 200444 P. R. China xujiaqiang@shu.edu.cn; State Key Laboratory of Transducer Technology, Shanghai Institute of Microsystem and Information Technology, Chinese Academy of Science Shanghai 200050 P. R. China jinqh@mail.sim.ac.cn; Faculty of Electrical Engineering and Computer Science, Ningbo University Ningbo 315211 P. R. China; College of Sciences, Shanghai Institute of Technology Shanghai 201418 P. R. China

## Abstract

Total phosphorus is one of the key water quality parameters in environmental monitoring. To precisely determine the total phosphorus, water samples have to be pretreated to convert the various forms of phosphorus to orthophosphate. Conventionally, pretreatment is accomplished by heating, acidification, and oxidation in a digestion equipment, which is dangerous, time-consuming, and complicated. Herein, we propose a novel high-performance electrochemical oxidation protocol for phosphorus digestion based on a PbO_2_ electrode. The electrode, which has a hydrophobic and stable surface, was prepared by electrochemical deposition on a titanium substrate and has high hydroxyl radical utilization when digesting total phosphorus. As a result, 90% of sodium glycerophosphate was digested within 30 minutes, and high digestion ratios of acephate, glyphosate, and inland water samples were obtained as well. In addition, this electrochemical digestion protocol does not required heating and acidification steps, which shortens the digestion time. Therefore, a rapid quantification of total phosphorus in the water sample was achieved.

## Abstract 2

Using electrochemical oxidation for digestion when testing total phosphorus with a digestion ratio of about 90%.

## Graphical abstract

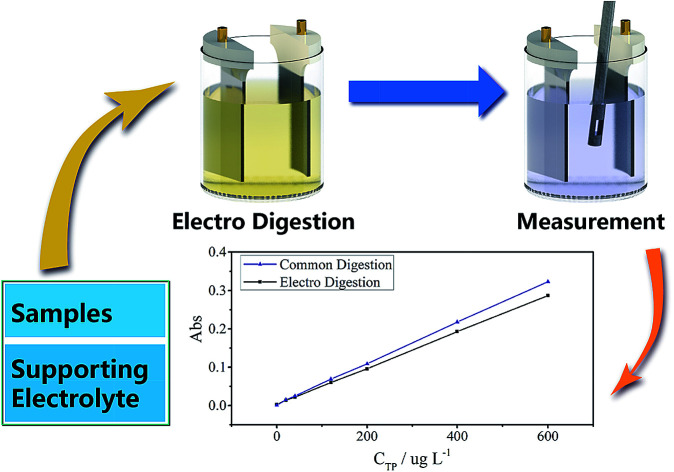

## Introduction

Phosphorus is a critical nutrient in nature, but excess amounts may cause eutrophication in lakes and upset the balance of an ecosystem.^1^ Eutrophication is a serious environmental problem that causes explosions in the growth of plants and algae. Their huge oxygen consumption may result in the depletion of oxygen in a water body, which is crucial for fish and other aquatic species, and the lack of dissolved oxygen may lead to their perishing.^2–6^ Many derived forms of phosphorus, which is a key nutrient of plankton, come from human excreta, agricultural runoff, industrial wastes, and other materials. In fact, eutrophication may be more severe in areas where the ratio of nitrogen to phosphorus is high, and it is possible that plankton, such as blue-green algae, may bring enough nitrogen into the biological cycle to balance the surplus of phosphate.^7^ However, the biogeochemical cycle of phosphorus is vast but very slow.^8,9^ Phosphorus is the primary limiting nutrient, and when it is replete, the biovolume of all cyanobacterial taxa probably increases with rising concentrations of P.^10–12^ However, the recovery of phosphorus from waste materials is difficult and still underdeveloped,^13,14^ therefore, the quantification of total phosphorus is important.

In natural water, phosphorus is present in a wide variety of chemical forms that can be separated in three broad classes: orthophosphates,^15^ condensed phosphates (pyro-, *meta*-, and poly-), and organic phosphorus. Total phosphorus (TP) is the sum of all forms of phosphates obtained by digestion and converted to orthophosphates.^16,17^ The analytical determination of TP is based on an ammonium molybdate spectrophotometric method, as described by the Chinese National Standard,^18^ which involves two main progresses. (1) The digestion of water; this approach converts all forms of phosphorus to orthophosphate. (2) The measurement of orthophosphorous; this approach involves a colorimetric analysis of phosphomolybdenum blue,^18–20^ and ammonium molybdate, ascorbic acid, and antimony potassium are added to form the blue colored a-keggin anion, ammonium phosphomolybdate. The reaction is as follows (eqn (1)):

1 7PO_4_^3−^ + 12Mo_7_O_24_^6−^ + 72H^+^ → 7PMo_12_O_40_^3−^ + 36H_2_O

The concentration of orthophosphate is proportional to the color of the samples and is determined by spectroscopy at 700 nm.^8^ High temperature and a strong oxidant are required in the digestion progress, which corresponds to the abovementioned step (1) and is called the pre-treatment. The water samples have to be heated to 120 °C for 30 minutes with an oxidizing agent, which is therefore energy and time consuming, requiring at least 3 hours for the pretreatment of the samples. The oxidative digestion procedure includes perchloric acid, hydrogen peroxide, sulfuric acid, nitric acid and peroxydisulfate,^21^ which make the analytical determination even more complex.^22^

2 PbO_2_ + H_2_O → PbO_2_(˙OH) + H^+^ + e^−^

Electrooxidation as a means to remove organic waste products such as methamidophos, chlorophenol and phenol from water has been widely reported with a good removal ratio for all three cited examples.^23–26^ Lead dioxide (β-PbO_2_) has been used as an electrode material with high oxidation power to degrade wastewater;^27–29^ when doped with fluoride, the performance of the β-PbO_2_ electrode showed a significant improvement.^30,31^ The oxidation of the β-PbO_2_ electrode is powered by hydroxyl radicals (˙OH) (eqn (2)). The corresponding mechanisms have been elucidated and show the possibility of oxidizing many forms of phosphorus.^32–34^ Consequently, to improve the digestion procedure to determine TP, we establish herein a rapid and energy-saving method using an advanced oxidation process to generate hydroxyl radicals (˙OH) with a β-PbO_2_ electrode, which shows a high hydroxyl radical utilization.^35^ ˙OH is one of the most powerful oxidizing agents, and the standard electrode potential is about 2.73 *vs.* SHE/V.^36,37^ A PbO_2_ electrode is used in this study to generate ˙OH with high-efficiency; it is an appropriate anode with effective electrocatalytic performances, a hydrophobic and stable surface, and low cost. Although boron-doped diamond film (BDD) electrodes have a higher OEP and better oxidation performance, its preparation is complicated and costly. In addition, the resistance of the BDD electrode is too high, and a high output potential has to be applied.^31^ ˙OH are composed of a hydrogen atom bonded to an oxygen atom, which makes them highly reactive, readily stealing hydrogen atoms from other molecules to form water molecules.^33^

Herein, the advanced oxidation process (AOP) applied to digest phosphorus samples using a PbO_2_ electrode for oxidation simplified and shortened the testing time to determine the total phosphorus. Sodium glycerophosphate, acephate, glyphosate, and adenosine triphosphate were used as simulated samples to evaluate our proposed method, and three inland water samples were collected for comparison between standard digestion and electro digestion.

## Experimental

### Apparatus and reagents

All chemicals used were obtained from Sinopharm Chemical Reagent, Shanghai Aladdin Biochemical Technology, Sigma-Aldrich and J&K Scientific. Colorimetric measurements were performed on a fiber spectrometer purchased from Ocean Optics; the light source was DT-MINI-2-GS, and the spectrometer was a Maya2000 Pro. The electrochemical workstation used in this study was a Gamry Interface 1000 (Gamry Instruments, USA). The structure of the obtained samples was checked using a DX-2700 type powder X-ray diffractometer. Micrographs of the PbO_2_ electrode were obtained with a JSM-7800F field emission scanning electron microscope (SEM).

### Preparation of the PbO_2_ electrode

The electrode was prepared by two successive procedures for layers of the alpha and beta phase of lead dioxide. The titanium plates (1 cm × 2.5 cm, 1.0 mm thickness) used as substrates were polished in advance using abrasive paper and then rinsed with a solution that contained 2.5 vol% HF and 20 vol% nitric acid for several seconds. After that, the plates were washed with acetone and deionized distilled water (DDW) in sequence before drying in flowing N_2_ stream. Electrochemical deposition of α-PbO_2_ was carried out under a potential of 1.8 V and current of 60 mA cm^−2^ in 0.11 mol L^−1^ PbO + 3.5 mol L^−1^ NaOH, and the solution was mixed by a magnetic stirrer. The electrode was then coated with a resin by soaking in a solution that contained HNO_3_ (0.1 mol L^−1^), Pb(NO_3_)_2_ (1 mol L^−1^), KF (0.035 mol L^−1^), and fluorine resin (10 ml L^−1^) under a potential of 2.7 V and current of 180 mA cm^−2^ to generate the β-PbO_2_ layer. The two-deposition progress was temperature-controlled at 65 °C, and the distance between the working electrode and the counter electrode was 1 cm.

### Digestion of samples

In the standard digestion procedure according to Chinese Nation Standard (GB 11893-89), 2 mL potassium persulfate solution (50 g L^−1^) were added into colorimeter tubes with a 12.5 mL water sample. The tubes were sealed and heated to 120 °C and were maintained at this temperature for 30 minutes, before cooling to room temperature and diluted to 25 mL using deionized water. The electrochemical digestion procedure was carried out galvanostatically at a current of 100 mA cm^−2^ with two electrodes on a 5 mL water sample to which 5 mL potassium sulfate solution (26 g L^−1^) was added and which was diluted to 25 mL using deionized water. Addition of potassium sulfate solution allowed adjusting of the ionic strength. Different electrochemical digestion times were studied, and the dilution ratio was varied in the two procedures so that the concentration of phosphate was equivalent.

### Measurement of orthophosphate

Colorimetric measurements were performed according to the Standard. 1 mL ascorbic acid solution (100 g L^−1^) and 2 mL molybdate solution (26 g L^−1^ ammonium molybdate tetrahydrate, 0.7 g L^−1^ antimony potassium tartrate and 1 mol L^−1^ 98% sulfuric acid) were added at an interval of 30 s. The absorbance of the solutions was measured at 700 nm after 5 minutes.

Pesticides containing phosphorus were purchased from the market, and inland water samples used as real samples were collected from the Suzhou River (Shanghai, China). According to the GB 11893-89, the total phosphorus in this study is reported as phosphorus P.

## Results and discussion

### Characterization of the prepared PbO_2_ electrode

**Fig. 1:**
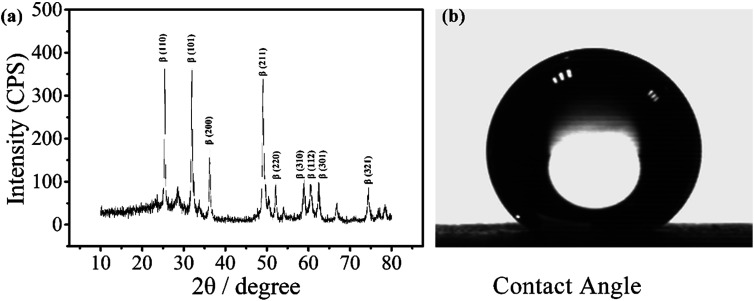
XRD pattern of a PbO_2_ electrode for electrochemical digestion and contact angle of the electrode.

The electrode was prepared by electrodepositing two layers of PbO_2_. The α-PbO_2_ layer was first electrodeposited in an alkaline solution containing NaOH and PbO. β-PbO_2_ was subsequently deposited in a lead nitrate solution. The electrode thus obtained was a polycrystalline deposit composed of a mixture of α- and β-phases with the predominance of the latter phase.^38^ As can be seen from the X-ray diffraction (XRD) patterns (Fig. 1(a)), the main orientations of the β-phases are (110), (101), (200), (211), and (110), (111), (002), (021) for the α-phases.^39^ Under normal conditions, β-PbO_2_ is the more stable polymorph, but its film is fragile and may fall off the substrate. Therefore, a α-PbO_2_ layer was used as a cushion.

The contact angle of the electrode was measured to be around 147°, as shown in Fig. 1(b), indicating that the material is highly hydrophobic. It is speculated that the PbO_2_ electrode with a hydrophobic surface doped with a fluorine resin may reduce the adsorption of ˙OH and improve its utilization.^31^

**Fig. 2:**
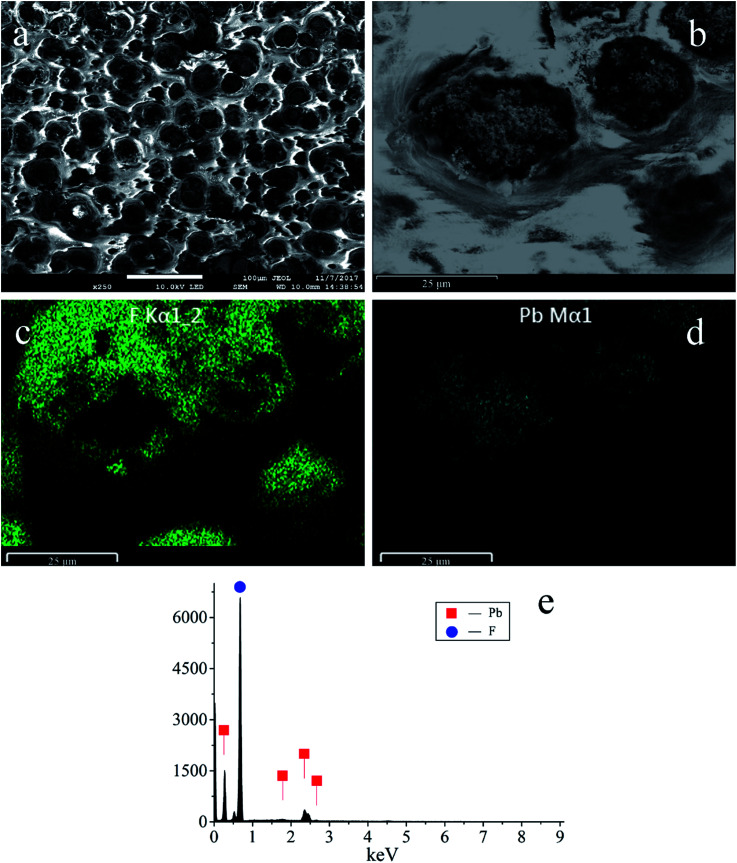
(a) and (b) SEM micrographs of the PbO_2_ electrode. (c), (d) and (e) EDS elemental mappings and EDS spectrum of F and Pb.

SEM micrographs were obtained and show a uniform distribution of the PbO_2_ particles and fluorine resin layer (Fig. 2). The diameter of the PbO_2_ particles ranges from 15 μm to 30 μm. The bright and smooth areas correspond to the fluorine resin deposits, which surround the PbO_2_ particles. The EDS elemental mapping of F is shown in Fig. 2(c), and the mapping of Pb is shown in Fig. 2(d). Their distribution highly coincides with the enlarged image (Fig. 2(b)). Fig. 2(e) shows the EDS spectrum of the electrode, and the weight percentages of Pb and F are about 22.82 wt% and 77.18 wt%, respectively.

**Fig. 3:**
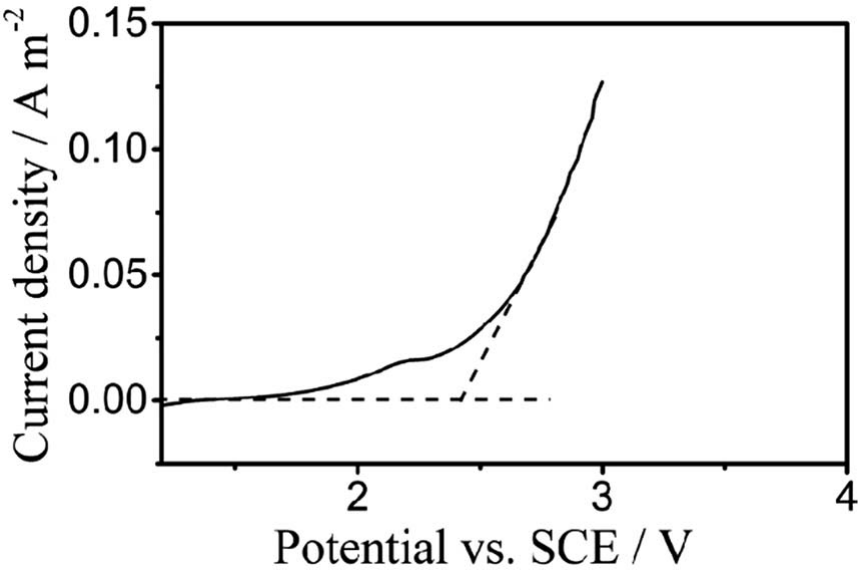
Polarization curves of the PbO_2_ electrodes for electrochemical digestion in 0.5 mol L^−1^ H_2_SO_4_ solution at a scan rate of 5 mV s^−1^.

Fig. 3 displays the liner polarization curves of the PbO_2_ electrode prepared for the digestion of TP in 0.5 mol L^−1^ H_2_SO_4_ solution at a scan rate of 5 mV s^−1^, where the OEP of the electrode can be observed at around 2.38 *vs.* SCE/V.^23,40^ The oxygen evolution potential (OEP) is an important feature for the application of anodes,^41^ and the high OEP of the electrode represents a high ˙OH yield, which reveals its ability to degrade organic compounds in aqueous solution.

### Electrochemical digestion of simulated samples

**Fig. 4:**
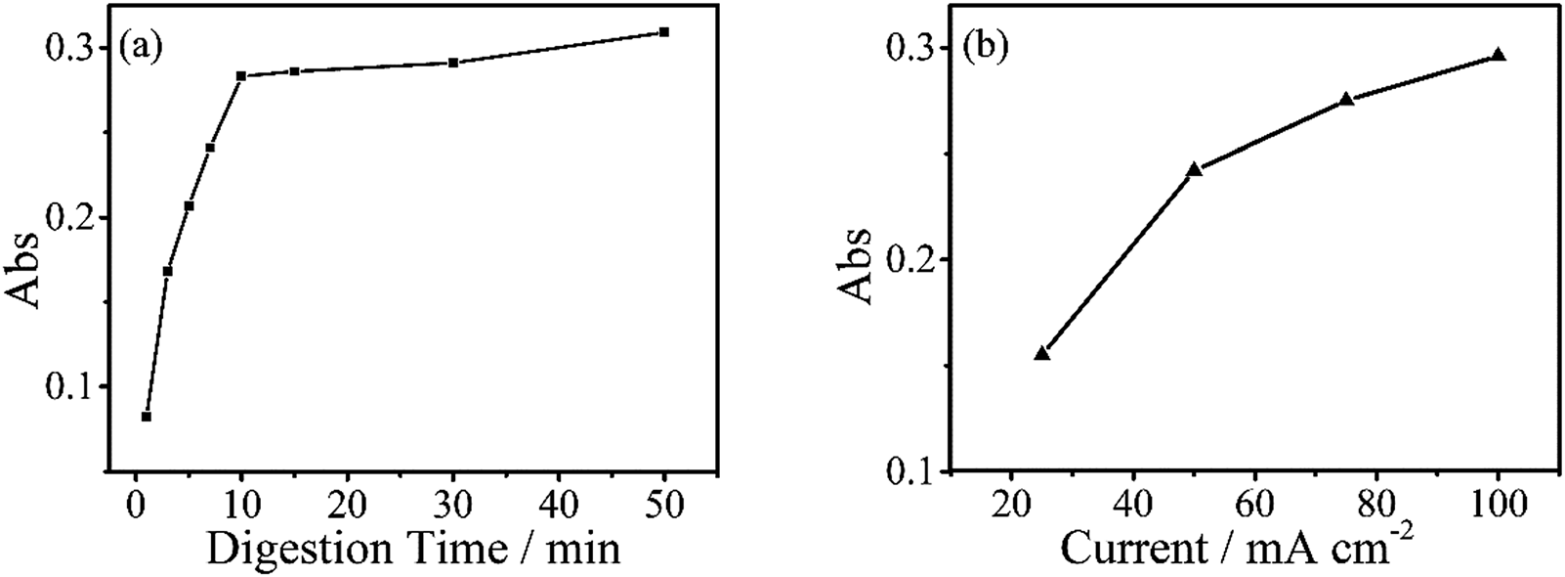
(a) Absorbance values of digested sodium glycerophosphate containing 600 μg L^−1^ of phosphorus with different digestion times, absorbance scarcely increases after 20 minutes. The digestions were performed at a current of 100 mA cm^−2^. (b) Digestion of same concentrations of sodium glycerophosphate solutions for 30 minutes with different currents.

The digestion of 600 μg L^−1^ sodium glycerophosphate solution containing 5.1 g L^−1^ potassium sulphate as supporting electrolyte for 30 minutes at different operating currents is shown in Fig. 4(b). The absorbance at 700 nm increases with the operating current albeit at a slower rate when the current exceed 150 mA cm^−2^. Because of the limitations of the electrochemical workstation, the final operating current was set to 100 mA cm^−2^ for subsequent measurements in this study. The evolution of the digestion time of the aqueous solutions is also depicted in Fig. 4(a), and the absorbance scarcely increases after 20 minutes. To achieve the highest ratio and the smallest digestion time, we set the reaction time to 30 minutes, although the digestion ratio kept rising until 50 minutes.

**Fig. 5:**
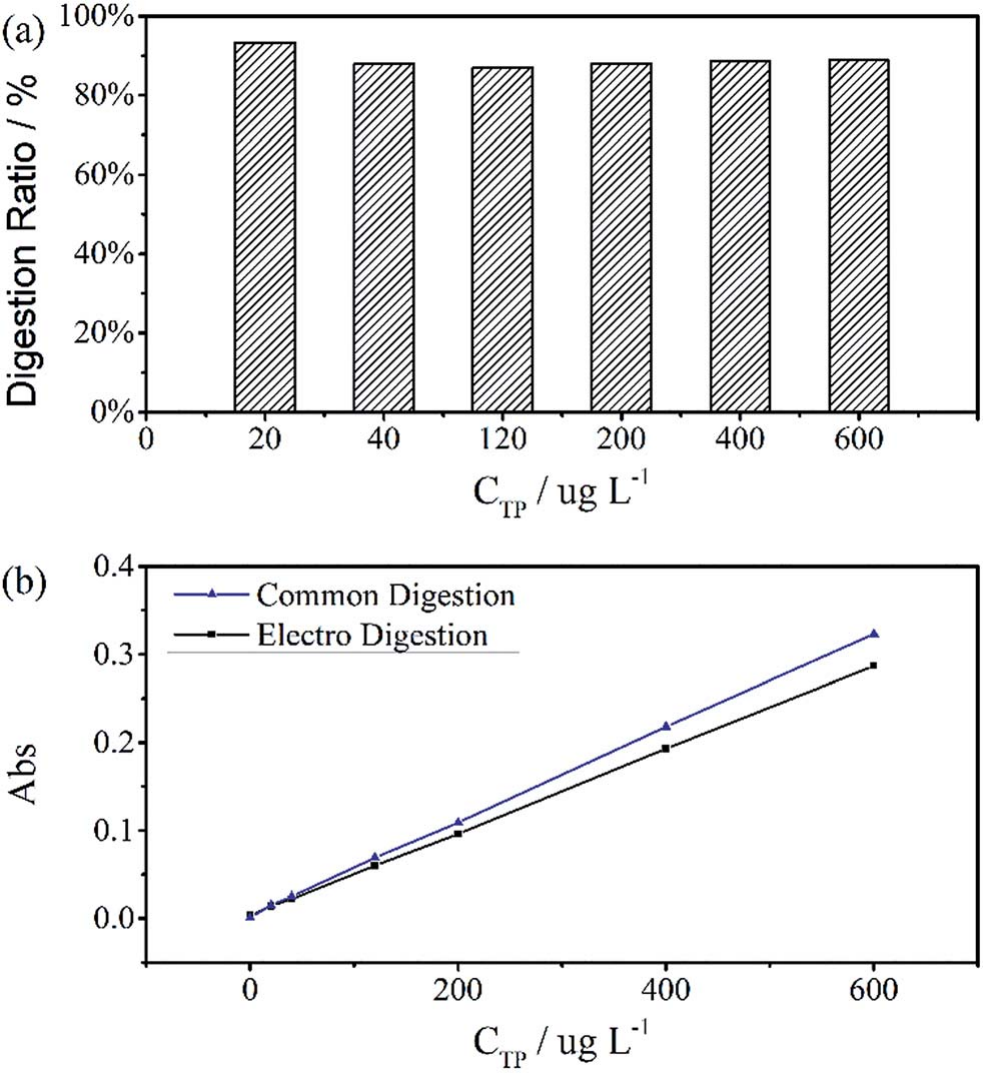
(a) Digestion ratios between standard digestion and electro digestion. Values are around 90%, the highest is 93.3% and lowest is 88.5%. (b) Two calibration curves revealing a perfect linear relationship, with *R*^2^ = 0.9998 for Standard Digestion and 0.9999 for electro digestion. The digestion time of samples was 30 minutes at a current of 100 mA cm^−2^.

A standard curve with minimal *R*^2^ of 0.999 was obtained for the electrochemical digestion, as shown in Fig. 5(b). To compare the two digestion methods, which are the standard digestion (according to Chinese National Standard) and the electrochemical digestion, the amount of TP determined from the absorbance value of sodium glycerophosphate was measured by spectrophotometry, and they present a good linearity. In this test, the samples were digested sequentially from low to high concentrations to avoid interference when the concentration of TP was too low. However, the real samples can each use one electrode. When the standard digestion is applied, potassium dihydrogen phosphate, which is used as the standard sample in the TP measurement, needs to be digested probably because of silicon molybdenum blue interference, silicon being separated from the colorimeter tube at high temperature. However, in electrochemical digestion, heat is not required and the potassium dihydrogen phosphate standard can be used directly. Electrochemical digestion ratios are calculated and depicted in Fig. 5(a). Most values lie around 90% (*C*_electrochemical_/*C*_standard_), with a highest value of 93.3% and lowest value of 88.5%, indicating that the digestion method is effective. The ratios are also stable within the range limit.

**Scheme 1:**
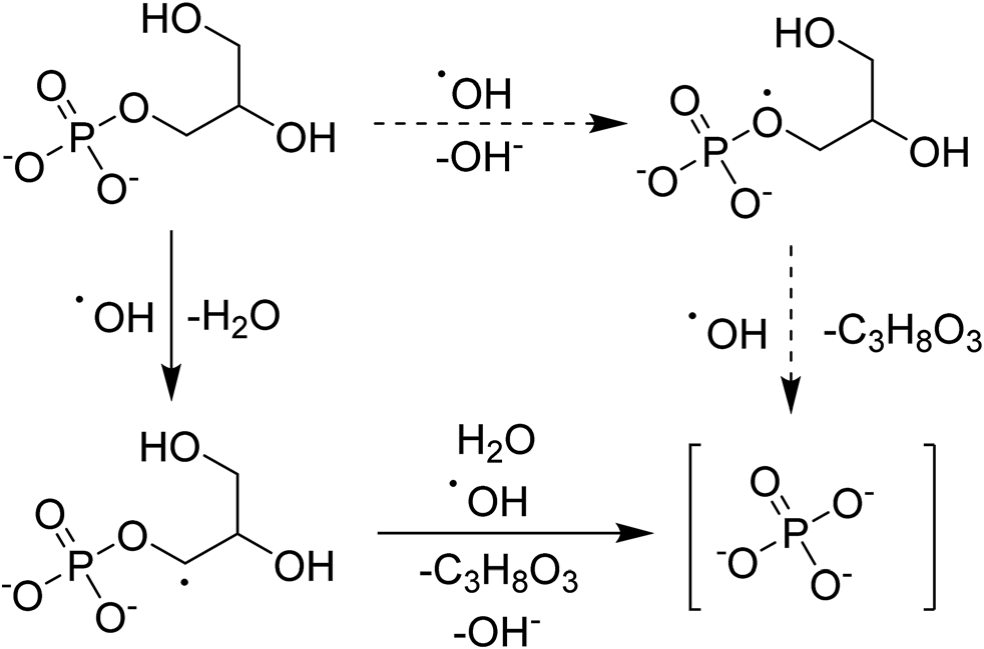
Hypothesized mechanism for the electrochemical digestion of sodium glycerophosphate.

The hypothesized mechanism for the electrochemical digestion of sodium glycerophosphate is shown in Scheme 1. The digestion induced by hydroxyl radicals may undergo an electron-transfer mechanism (solid arrows) or an H abstraction mechanism (dashed arrows); then, orthophosphate and glycerin are produced.^42–44^

**Fig. 6:**
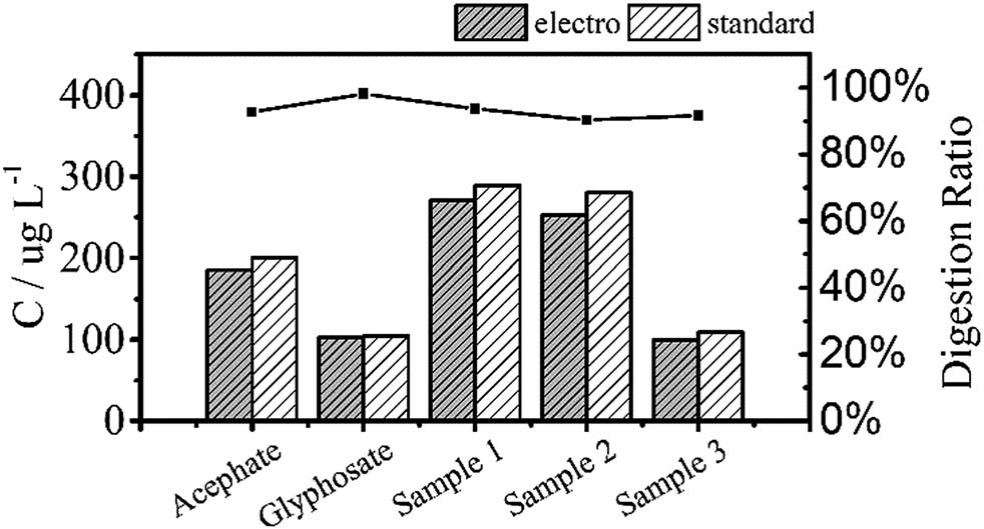
Total phosphorus in pesticide and inland water samples that were used as real samples measured by two digestion methods. All digestion ratios exceed 90%.

Fig. 6 shows the TP measurements from the digestion of the common phosphorus-containing pesticides (acephate, glyphosate) used as simulated samples and inland water used as real samples. The black area results from electrochemical digestion and the white area corresponds to standard digestion, and both show similar testing results. The electrochemical digestions are performed at a current of 100 mA cm^−2^ for 30 minutes, similarly to the above tests, and 12.5 mL of the real sample containing 5 mL potassium sulphate was diluted to 25 mL in each test. There are some differences in the digestion ratios of the real samples, which are probably due to suspended solids that adsorb small amounts of the total phosphorus, which is difficult to digest by the electrochemical method.

## Conclusions

An electrochemical digestion method for measuring total phosphorus in simulated and real samples using a PbO_2_ electrode to generate ˙OH as the oxidizing agent has been reported. The proposed electrochemical digestion procedure is simple, fast and energy-saving, and the ultimate measurement results show a good digestion ratio of about 90%, which remained stable in the measuring range. Electrochemical digestion and standard digestion correlate linearly in TP tests. Further applications of this electrochemical digestion approach are under development.

## Conflicts of interest

There are no conflicts to declare.

## Supplementary Material
